# *PTPN22* 1858C>T gene polymorphism in patients with SLE: association with serological and clinical results

**DOI:** 10.1007/s11033-014-3498-6

**Published:** 2014-07-02

**Authors:** Lidia Ostanek, Magdalena Ostanek-Pańka, Danuta Bobrowska-Snarska, Agnieszka Bińczak-Kuleta, Katarzyna Fischer, Mariusz Kaczmarczyk, Andrzej Ciechanowicz, Marek Brzosko

**Affiliations:** 1 Department of Rheumatology and Internal Medicine, Pomeranian Medical University, Szczecin, Poland; 2 Department of Clinical and Molecular Biochemistry, Pomeranian Medical University, Szczecin, Poland

**Keywords:** *PTPN22* gene polymorphism, Systemic lupus erythematosus, Antiphospholipid antibodies, Antiphospholipid syndrome

## Abstract

To assess the association between *PTPN22* 1858C>T gene polymorphism and susceptibility to, and clinical presentation of, systemic lupus erythematosus (SLE). Our study included 135 SLE patients (120 women and 15 men; mean age 45.1 years; mean course of disease from 0.5 to 31 years) and 201 healthy subjects. The *PTPN22* 1858C>T gene polymorphism was genotyped by polymerase chain reaction restriction fragment length polymorphism. A significantly higher incidence of genotype CT in patients with SLE (36.3 %) was found, compared with the control group (24.9 %). The frequencies of C1858 and T1858 alleles were 78.1 and 21.9 % in SLE patients and 86.1 and 13.9 % in controls, respectively. Significantly higher SLE susceptibility was observed in patients carrying at least one T allele (*p* = 0.009; OR 1.86; 95 % CI 0.14–3.05). Significant association of the *PTPN22* T1858 allele (CT + TT vs.CC) and secondary antiphospholipid syndrome was observed (*p* = 0.049). In SLE patients carrying the T1858 allele, higher levels of antiphospholipid antibodies (anticardiolipin antibodies and/or lupus anticoagulant) were found (*p* = 0.030; OR 2.17; 95 % CI 1.07–4.44).

## Introduction

Systemic lupus erythematosus (SLE) is a systemic disease of connective tissue, with defective regulation of immune responses as a leading feature in its pathogenesis, which ultimately leads to the development of inflammation and damage to many organs and tissues. The reasons for the development of SLE are not fully understood, but a growing body of research suggests that genetic mechanisms are involved in combination with environmental factors.

*PTPN22* appears to be the second most important genetic risk factor for autoimmune diseases, following the HLA system [1, 2]. It is located on chromosome 1 (1p13.1–lp13.3) and is responsible for encoding the cytoplasmic tyrosine phosphatase (synonym lymphoid tyrosine phosphatase; Lyp). It is assumed that Lyp, together with Csk (which is a tyrosine protein kinase encoded by the CSK gene), prevent spontaneous activation of T cells. 1858C>T *PTPN22* gene polymorphism affects the binding of Lyp to Csk. Replacing arginine (R) by tryptophan (W) is the cause of an abnormal interaction between Lyp and Csk, leading to abnormal regulation of T cells by kinases such as Lck, Fyn and/or ZAP-70 [3].

The identified association between the *PTPN22* 1858C>T gene polymorphism and T cell activation suggests that it may contribute to development of autoimmune diseases [4]. A relationship between the presence of the 1858T allele and the prevalence of diabetes mellitus type I has been shown. The same allele has also been associated with susceptibility to rheumatoid arthritis, Graves and Hashimoto’s disease [5]. Some researchers believe that *PTPN22* gene polymorphism is involved in pathogenesis of the familial, non-sporadic occurrence of SLE [6].

The aim of our study was:

1. To evaluate association between *PTPN22* 1858C>T polymorphism and susceptibility to systemic lupus erythematosus.
2. To show whether *PTPN22* 1858C>T polymorphism influences the clinical course of systemic lupus erythematosus.
3. To assess association between *PTPN22* 1858C>T polymorphism and the presence of specific autoantibodies.

## Materials and methods

The study comprised 135 patients with SLE (120 females and 15 males), hospitalized at the Department of Rheumatology and Internal Medicine, Pomeranian Medical University, Szczecin, from 1996 to 2002, or staying under Outpatient Rheumatology Clinic. The diagnosis of SLE was established according to the modified ARA criteria from 1982 [7]. The average age of patients was 45.1 ± 13.6 years (y); disease duration from 0.5 to 31 y, mean 8.2 ± 7.2 y.

The control group consisted of 201 samples of genomic DNA (84 females and 117 males) isolated from umbilical cord blood from healthy, consecutively born infants who were born after 37 completed weeks of pregnancy. These samples came from the DNA bank at the Department of Clinical and Molecular Biochemistry, Pomeranian Medical University, Szczecin, Poland. Studies where newborns are used as a control group have advantages because environmental influences such as: diet, lifestyle, smoking and associated diseases are reduced [8]. The control group consisted of consecutive healthy newborns. It is a sample of homogenous healthy population from the North-Western part of Poland, the region where the study group comes from. The choice of the random control group creates the most optimal comparator to estimate allele frequency in the general population. The use of unscreened controls have been previously investigated as a tool in case–control studies by Moskvina et al. [9].

All patients in the study and control groups were of European descent. Consent from the Bioethics Committee at the Pomeranian Medical University (BN-001/57/05, dated 21st of February, 2005), and patients’ informed consent for cases as well as parental informed consent for controls, were obtained.

Analysis of medical histories focused on age at onset and the nature of initial symptoms of the disease, i.e. symptoms up to the time of diagnosis, followed by symptoms that occurred at diagnosis and during observation. We have thoroughly analyzed the whole course and pregnancy termination in females SLE patients. During physical examinations of patients, particular attention was paid to the symptoms associated with SLE (according to SLE criteria) and symptoms that may result from the presence of anti-phospholipid antibodies (aPL) e.g. thrombosis of veins and arteries, livedo reticularis, Raynaud’s phenomenon, thrombocytopenic bleeding disorder, symptoms of nervous system involvement, and cardiovascular diseases. SLE activity was assessed using the Systemic Lupus Erythematosus Activity Index (SLEDAI) [10]. Activity was considered as low with a SLEDAI score from 0 to 11; moderate activity: 12–20; and high activity: more than 20.

### Identification of *PTPN22* 1858C>T gene polymorphism

Genomic DNA was extracted from 0.15 mL of K_3_EDTA-anticoagulated blood with a QIAamp DNA Mini Kit (QIAGEN) and was amplified by PCR with primers flanking the C1858T (rs2476601) polymorphic region *PTPN22*: 5′- ACCGCGCCCAGCCCTACTTTTG -3′ as sense primer, and 5′- AGCCACCATGCCCATCCCACACT -3′ as anti-sense primer. The reaction was carried out in a total volume of 20 μL containing: 40 ng of template DNA, 4 pM of each primer, 1 × PCR buffer—combination of KCl and (NH_4_)_2_SO_4_, (exact concentrations patent protected) (QIAGEN), 1 × Q-Solution—PCR additive that facilitates amplification of difficult templates by modifying the melting behavior of DNA (composition of the mixture patent protected) (QIAGEN), 1.5 mM MgCl_2_ (QIAGEN), 200 μM each dNTP (MBI Fermentas) and 1 U of HotStarTaq polymerase (QIAGEN).

The amplification was performed with initial denaturation at 94 °C for 15 min, and then 37 cycles: denaturation at 94 °C for 20 s, annealing at 60 °C for 40 s, and extension at 72 °C for 40 s. The final 72 °C incubation was extended by 8 min. For restriction fragment length polymorphism assays, an 8.5 μL aliquot of PCR product was incubated at 37 °C for 12 h with 5 U *Rsa* I (Rhodopseudomonas sphaeroides, 5′-GT↓AC-3′) (MBI Fermentas, Lithuania).

Fragments were separated by electrophoresis on 3 % agarose gels, stained with ethidium bromide. Results were recorded with photographs of gels using UV light.

### Statistical analyses

Statistical analyses were performed using the χ^2^ or Fisher’s exact test and unpaired Student’s *t* test, for discontinuous and continuous variables, respectively. Fisher’s exact test was applied to test for Hardy–Weinberg proportion. Odds Ratios (OR) and 95 % confidence intervals (95 % CI) were calculated using logistic regression models. Calculations were carried out using the statistical software STATISTICA [StatSoft, Inc. (2011), version 10, www.statsoft.com] and package “genetics” for R software (http://cran.r-project.org). A *p* value <0.05 was considered statistically significant.

## Results

**Fig. 1:**
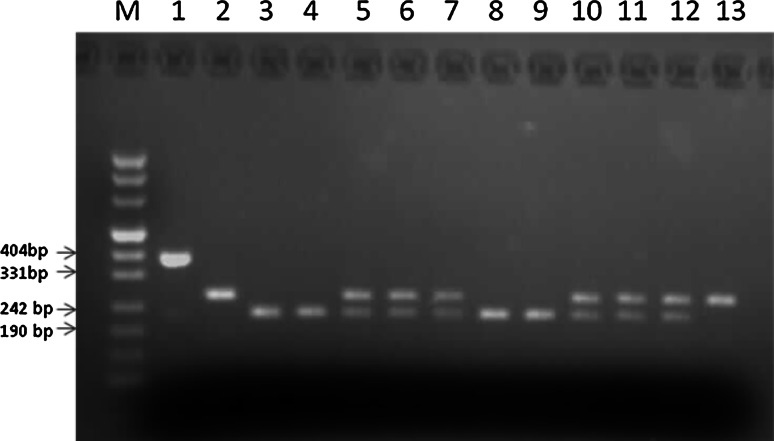
Identyfication of rs2476601 *PTPN22* gene polymorphism. *Lanes M* DNA length marker (Puc Mix Marker 8, MBI Fermentas), *1* uncleaved amplification product, *2*, *13* TT homozygotes, *3*, *4*, *8*, *9* CC homozygotes, *5*, *6*, *7*, *10*, *11*, *12* CT heterozygotes

Amplification gave products of 392 bp in length. The PCR product from a C1858 allele was cleaved into 228, 74, 46 and 44 bp fragments. A T1858 allele was cleaved into 272, 74 and 46 bp fragments (Fig. 1).

The study groups, both SLE-patients and controls, were in Hardy–Weinberg equilibrium (Table 1).

**Table 1:** Frequency distributions of *PTPN22* genotypes and alleles in SLE-patients and controls

Genotypes	SLE, n (%)	Controls, n (%)	*p* (χ^2^ test)	OR (95 % CI)
CC	81 (60.0)	148 (73.6)	–	1.00
CT	49 (36.3)	50 (24.9)	0.016*	1.79 (1.08–2.97)
TT	5 (3.7)	3 (1.5)	0.117†,*	3.05 (0.61–16.55)
CT + TT	54 (40.0)	53 (26.4)	0.009*	1.86 (1.14–3.05)
*Allele*				
C	211 (78.1)	346 (86.1)	0.008	1.73 (1.13–2.64)
T	59 (21.9)	56 (13.9)		
All genotypes, n	135	201		
Hardy–Weinberg equilibrium	0.596	0.508		

* Versus CC; *p* = 0.024 (χ^2^ test) for CC versus CT versus TT

† Fisher’s exact test

The distribution of genotypes and alleles in patients with SLE was significantly different compared to the control group (*p* = 0.024 and *p* = 0.008, respectively). A significantly higher incidence of genotype CT was found in patients with SLE (36.3 %), compared with the control group (24.9 %). Genotype CC in patients with SLE occurred significantly less frequently than in the control group. Carriage of at least one T allele (CT + TT vs. CC) significantly increased the risk of developing SLE (*p* = 0.009; OR 1.86; 95 % CI 1.14–3.05). The T1858 allele frequency in patients with SLE was 21.9 %, which was significantly higher than in the control group, where it was found to be 13.9 % (Table 1).

No association between gender and genotype in the whole group, SLE patients and control group have been found (*p* = 0.081, *p* = 0.613, *p* = 0.219 respectively) (Table 2).

**Table 2:** *PTPN22* 1858C>T gene polymorphism in SLE patients and controls with regard to gender

Genotypes	SLE + Controls		SLE		Controls	
	Males n (%)	Females n (%)	Males n (%)	Females n (%)	Males n (%)	Females n (%)
CC	98 (74.2)	130 (64.0)	10 (66.7)	70 (58.8)	88 (75.2)	60 (71.4)
CT	30 (22.7)	69 (34.0)	4 (26.7)	45 (37.8)	26 (22.2)	24 (28.6)
TT	4 (3.0)	4 (2.0)	1 (6.7)	4 (3.4)	3 (2.6)	0 (0)
*p* (χ^2^ test)	0.081		0.613		0.219	

Analysis of the relationship between specific genotypes with clinical symptoms of SLE showed significantly higher incidence of the T1858 allele (CT + TT vs. CC) in patients with secondary antiphospholipid syndrome (SAPS) (Table 3). This association was not observed in relation to other clinical manifestations.

**Table 3:** Clinical characteristics of SLE patients in regard to *PTPN22* 1858C>T gene polymorphism

Clinical manifestations	Genotypes		*p*†
	C/C (81) n (%)	C/T + T/T (54) n (%)	
Skin involvement	45 (55.6)	31 (57.4)	0.480
Photosensitivity	45 (55.6)	22 (40.7)	0.184
Oral ulcers	17 (21.0)	8 (14.8)	0.462
Arthritis	73 (90.1)	43 (79.6)	0.380
Serositis	25 (30.9)	20 (37.0)	0.291
Renal disorder	19 (23.5)	19 (35.2)	0.076
Neurologic disorder	40 (58.8)	28 (41.2)	0.473
Leucopenia	42 (60.9)	27 (39.1)	0.776
Lymphopenia	32 (57.1)	24 (42.9)	0.609
Thrombocytopenia	18 (52.9)	16 (47.1)	0.409
SAPS	24 (29.6)	25 (46.3)	0.0485*
Vasculitis	17 (21.0)	5 (9.3)	0.102
Ischemic heart disease	9 (11.1)	9 (16.7)	0.248
Adverse pregnancy outcome in anamnese	10 (45.5)	12 (54.6)	0.085

† Student’s test

* Statistically significant

High level of anticardiolipin antibodies or (lupus anticoagulant) LA occurrence were more common in the group of SLE patients carrying the CT or TT genotype (*p* = 0.030) (Table 4) with OR 2.17; 95 % CI 1.072–4.437.

**Table 4:** Autoantibodies in SLE patients with regard to *PTPN22* 1858C>T gene polymorphism

Autoantibodies	No. of SLE patients with specific autoantibodies detected		*p*†
	Genotype C/C (total = 81) n (% of total)	Genotypes C/T + T/T (total = 54) n (% of total)	
Anti-dsDNA	28 (34.6)	21 (38.9)	0.49
Anti-nucleosome	26 (32.1)	13 (24.1)	0.25
Anti-histone	13 (16.1)	5 (9.3)	0.28
Anti-Sm	5 (6.2)	4 (7.4)	0.75
Anti cardiolipin IgG	50 (61.7)	28 (51.9)	0.31
Anti cardiolipin IgM	31 (38.3)	17 (31.5)	0.46
Anti cardiolipin IgG or IgM	38 (46.9)	33 (61.1)	0.08
Lupus anticoagulant	12 (14.8)	14 (25.9)	0.12
Anti β^2^GPI screen	18 (22.2)	15 (27.8)	0.37
Any aPL	32 (39.5)	32 (59.3)	0.03^*^

† χ2 test

* statistically significant

Any aPL: anticardiolipin IgG or IgM or lupus anticoagulant or anti β2GPI antibodies.

## Discussion

Genetic and environmental factors play an important role in the predisposition to autoimmune diseases. In the present study on patients with SLE, significantly higher incidence of the *PTPN22* 1858C>T allele was shown. In agreement with the results of studies by Eliopoulos et al. [11] conducted in the Caucasian population of Southern Europe, by Orozco et al. [12] in the Spanish population, as well as by Piotrowski et al. [13] in the Polish population, higher incidence of the T1858 allele and genotype CT was found. According to Piotrowski et al. [13], there was no increased risk of SLE in TT homozygous patients.

A few studies have not supported a putative association of *PTPN22* polymorphism with SLE [6, 14, 15]. Wu Hui et al. [16] did not observe this relationship in families of Caucasian patients with SLE living in North America, Finland and Great Britain, although in carriers of this polymorphism they found a higher risk of developing autoimmune thyroid disease. A study by Kaufman et al. [6], on Americans of European descent, clearly showed a higher incidence of T1858 allele of *PTPN22* gene in patients with familial but non-sporadic SLE. Interestingly, African Americans had a much lower prevalence of the T allele than Europeans and Hispanics [16, 17].

The presence of the T1858 allele of the *PTPN22* gene probably results in weaker binding of lymphocytic phosphatase with regulatory tyrosines, leading to over-reactivity of T cells, which underlies a resulting autoimmune process.

We have found a few reports suggesting association between *PTPN22* 1858C>T gene polymorphism and presence of autoantibodies in SLE patients. In our study the occurrence of antiphospholipid antibodies and SAPS in polish Caucasian SLE patients was assessed. The results of the study have shown a link between the presence of the *PTPN22* 1858C>T allele and the presence of specific antiphospholipid antibodies (aCL and/or LAC), and SAPS. We have not found other publications in which these parameters were assessed in the Polish population. Castro-Marrero et al. [18], in Spanish Caucasian patients did not find any association of *PTPN22* 1858C>T gene polymorphism with primary antiphospholipid syndrome. Kyogoku et al. [19] reported no association between the presence of the T1858 allele of the *PTPN22* gene with occurrence antinuclear antibodies (ANA) and aCL in a population of North American white individuals with SLE.

The incidence of genotypes CT and TT in patients with renal involvement in the course of SLE was shown by Reddy et al. [20], and this relationship was particularly pronounced in patients with *PTPN22*(+) PDCD1(-). This was also shown by Moez et al. [21], who evaluated the *PTPN22* 1858C>T gene polymorphism in a population of Egyptian patients with SLE. In the present study association between nephropathy and *PTPN22* 1858C>T gene polymorphism is at the limit of statistical significance. Additionally association between *PTPN22* 1858C>T gene polymorphism and adverse pregnancy outcome in the history gave the same statistical results. This could be related to the presence of antiphospholipid antibodies, which are well known as a risk factor for obstetric failures in SLE patients [22].

*PTPN22* 1858C>T gene polymorphism is the best genetic risk factor so far documented, independent of HLA, a for rheumatoid arthritis [23]. As Kariuki et al. [24] have shown, the presence of the 1858C>T variant is associated with abnormal serum cytokine profile in the form of high concentrations of IFNα and low concentrations of TNF, resulting in an increased risk of developing autoimmune diseases. Association of certain autoimmune disorders and T1858 allele of the *PTPN22* gene has been confirmed in many populations, although the T1858 allele frequencies vary considerably in different ethnic groups.

In conclusion, this study shows that the carriage of *PTPN22* T1858 allele predisposes to SLE, and it is associated with an increased risk of occurrence of antiphospholipid antibodies and secondary antiphospholipid syndrome.
